# Socioeconomic inequality and urban-rural disparity of antenatal care visits in Bangladesh: A trend and decomposition analysis

**DOI:** 10.1371/journal.pone.0301106

**Published:** 2024-03-25

**Authors:** Biplab Biswas, Nishith Kumar, Md. Matiur Rahaman, Sukanta Das, Md. Aminul Hoque

**Affiliations:** 1 Faculty of Science, Department of Statistics, Bangabandhu Sheikh Mujibur Rahman Science and Technology University, Gopalganj, Bangladesh; 2 Faculty of Science, Department of Statistics, University of Rajshahi, Rajshahi, Bangladesh; 3 Faculty of Science, Department of Statistics, Begum Rokeya University, Rangpur, Bangladesh; Khulna University, BANGLADESH

## Abstract

**Background:**

Socioeconomic inequality in antenatal care visits is a great concern in developing countries including Bangladesh; however, there is a scarcity of investigation to assess the factors of inequality and these changes over time. In this study, we investigated the trend of socioeconomic inequalities (2004–2017) in 1^+^ANC and 4^+^ANC visits, and extracted determinants contributions to the observed inequalities and urban-rural disparities in Bangladesh over the period from 2011 to 2017.

**Methods:**

The data from the Bangladesh Demographic and Health Surveys (BDHS) conducted in 2004, 2007, 2011 and 2017 were analyzed in this study. The analysis began with exploratory and bivariate analysis, followed by the application of logistic regression models. To measure the inequalities, the Erreygers concentration index was used, and regression-based decomposition analyses were utilized to unravel the determinant’s contribution to the observed inequalities. The Blinder-Oaxaca type decomposition is also used to decompose the urban-rural disparity into the factors.

**Results:**

Our analysis results showed that the prevalence of 1^+^ANC and 4^+^ANC visits has increased across all the determinants, although the rate of 4+ANC visits remains notably low. The magnitudes of socioeconomic inequality in 4^+^ANC visits represented an irregular pattern at both the national and urban levels, whereas it increased gradually in rural Bangladesh. However, inequalities in 1^+^ANC visits declined substantially after 2011 across the national, rural and urban areas of Bangladesh. Decomposition analyses have suggested that wealth status, women’s education, place of residence (only for 4^+^ANC visits), caesarean delivery, husband education, and watching television (TV) are the main determinants to attribute and changes in the level of inequality and urban-rural disparity between the years 2011 and 2017.

**Conclusions:**

According to the findings of our study, it is imperative for authorities to ensure antenatal care visits are more accessible for rural and underprivileged women. Additionally, should focus on delivering high-quality education, ensuring the completion of education, reducing income disparity as well as launching a program to enhance awareness about health facilities, and the impact of caesarean delivery.

## Introduction

Antenatal care (ANC) is a common and widely recognized process for protecting the health of mothers and their unborn infants [1]. Through ANC, pregnant women can learn about healthy behaviour, nutrient supplementation, pregnancy symptoms, tetanus immunization, and social and psychological support from skilled health workers [2]. To meet the third sustainable development goal (SDG3), Bangladesh has set a target of reducing maternal mortality ratio (MMR) to 105 per 100,000 live births in 2022 [3], with a global goal of reducing MMR to 70 per 100,000 live births by 2030 [4,5]. To accomplish this, maternal health care services including antenatal care visits are crucial, and play a vital role in decreasing maternal and child mortality rates [6]. According to the World Health Organization (WHO) [7], pregnant women need to receive at least four antenatal care visits (4^+^ANC) from trained medical professionals to reduce the complications associated with delivery. In Bangladesh, the utilization rate of at least one antenatal care visit (1^+^ANC) is significantly higher when compared to the utilization rate of 4^+^ANC visits [8]. However, there has been an increase in both 1^+^ANC visit rates (67.7–92.0%) and 4^+^ANC visit rates (25.5–47.0%) from 2011 to 2017. Despite this progress, the wealth-related gap among mothers is a barrier to accessing health services, particularly for ANC visits, and it is wider for 4^+^ANC visits. The gap between two extreme socioeconomic groups in respect of 1^+^ANC rate was 48.0–93.0% and 82.4–98.9% in 2011 and 2017, respectively [9,10]. Although there have been recent improvements in child and maternal health in Bangladesh [11], persistent inequalities in healthcare indicators still exist [12–15].

About 150 years ago, public health experts were concerned about the adverse effects on health due to the inequality among subgroups of the population [16]. Recent studies have demonstrated that health-related outcomes are concentrated in the extremely lower subgroups in the whole world [17–21]. Especially, the disparity in health outcomes between the wealthy and the impoverished is significant due to unequal access to healthcare facilities in low and middle-income countries [22–24]. As a result, socioeconomic inequalities and their impact on population health, as well as maternal and child health, have emerged as crucial topics over the past few decades [16–19]. Furthermore, the United Nations General Assembly emphasized reducing inequality to receive healthcare facilities for everyone in an opportunity [25]. Therefore, in Bangladesh, measuring and addressing the socioeconomic inequality in 1^+^ANC visits and 4^+^ANC visits are essential to ensure equal benefits for women of all socioeconomic backgrounds. It is also desired to identify the determinants and measure these contributions to the inequality in 1^+^ANC and 4^+^ANC using decomposition methods. To explain the associated determinants that contribute to the inequality and how changes the inequality due to contributing factors between two periods, an Oaxaca-type decomposition analysis is inevitable [26,27]. Moreover, decomposing the outcome variable (1^+^ANC and 4^+^ANC) in terms of inequality between two regional groups (rural and urban) may be reasonable to observe the real scenarios. This is accomplished through the Bilnder-Oaxaca decomposition method [28,29].

In Bangladesh, numerous studies have been carried out to assess the determinants of the number of antenatal care visits [30–32], particularly by taking at least four antenatal care visits suggested by WHO [13,33,34]. Rahman et al.[14] documented the trends, identified the determinants and computed the concentration index for at least 4 ANC visits using two BDHS data. Pulok et al. [12] demonstrated the socioeconomic inequality in maternal healthcare by considering the regional variation. Another study illustrated the inequality in maternal health care variables like antenatal care, postnatal care and skilled birth attendant etc. [35]. Even though there have been several studies on wealth-based inequality in health indicators and decomposing the inequality index into significant factors, only two studies have utilized the Oaxaca-type decomposition [26,27,36–38]. However, this method has not been applied to analyse ANC visits. A decomposition study was conducted to analyze the variations in socioeconomic inequality in delivery care services indicators such as facility delivery, skilled birth attendance, and C-section delivery [27]. Another decomposition analysis was carried out to demonstrate the role of various factors in the inequality of childhood morbidity [26]. However, these studies did not address certain methodological issues related to the decomposition and quantification of wealth-based inequality in health outcomes, particularly in binary health outcomes among regional and racial groups. Furthermore, we know so far from the literature review that the study on inequality in ANC using health inequality measures like concentration index and its decomposition was absent. Although, there exists a huge gap in receiving ANC visits between urban and rural women but the existing studies missed this issue. Hence, in this study, we have shown the trends, determinants contribution to the inequality during the period, and decomposing disparity on a regional basis for ANC visits. The objectives of this study are as follows: (i) to measure the socioeconomic inequality using the Erregers Concentration Index (ECI) for 1^+^ANC (Yes/No) and 4^+^ANC (<4, ≥4), (ii) to identify the factors associated with 1^+^ANC and 4^+^ANC, (iii) to fit a logistic regression model for the associated factors using the forward selection approach and to measure the marginal effects as well as elasticity (iv) to apply Wagstaff decomposition for measuring the contribution of affluent factors to the ECI and Oaxaca type decomposition are used to measure the changes (%) inequality between 2011 and 2017 and (v) to compare the contributions of associated factors of the disparities by mother’s place of residence (rural/urban) using the Blinder-Oaxaca decomposition.

## Methods

### Data and sample

The trend and decomposition analysis of socioeconomic inequality in antenatal care visits (yes/no) and at least four antenatal care visits with the associated determinants were analysed using the 2004, 2007, 2011, and 2017–2018 rounds of the Bangladesh Demographic and Health Surveys (BDHS) data [8,9,39]. Under the global DHS program, BDHS is a nationally representative cross-sectional survey conducted in every three years. To collect the mother’s health care and health-related information during their reproductive age span (15–49), a complex multistage sample design is employed. The response rate is about 98% in these surveys. We pre-process the datasets by removing missing, unusual and inconsistent observations (approximately 2%) on selected variables for the analysis. After all, 5364, 4763, 7178 and 4946 women were selected as our final sample in 2004, 2007, 2011 and 2017, respectively.

#### Response variable

The target variable is the number of ANC visits obtained from ever-married women who gave birth within three years before the surveys and were aged between 15–49 years. Questions about antenatal care visits and their probably associated socioeconomic and demographic variables were asked of the mothers. We have re-coded the target variable as a binary variable, 1^+^ANC visits denoting 1 if the number of ANC visits is at least one and 0 otherwise, 4^+^ANC visits denoting 1 if the number of ANC visits is at least four and 0 if the number of ANC visits is less than four.

#### Major explanatory variables

We selected explanatory variables based on the literature review and the information on BDHS datasets. About 20 household, individual, geographical and community-level explanatory variables were considered for the analysis and model building. Among these, women’s educational status, partner’s education level, last birth C-section, age of respondent, age at first birth, birth order, pregnancy wanted, respondent currently working, partner’s occupation, and respondent occupation were the potential individual-level factors and considered two geographical factors: division and place of residence (urban/rural). We define mass media exposure as "yes" if the household had access to any one of electronic media, broadcasting or newspaper, and “no” otherwise. This study determines the socioeconomic status of the individuals based on an asset-based wealth index. The wealth index was calculated using asset variables (such as household has TV/radio, refrigerator, sanitation and water facilities, materials of house construction, farming land etc.) through principal component analysis (PCA) techniques. Asset score is used to rank the household into five socioeconomic groups from poorest (lowest 20%) to richest (top 20%). We included these variables in the analysis to assess the degrees of inequality in response variables and their decomposition to explanatory variables from 2011 to 2017.

### Measurement and statistical analysis

We assessed the changes in 1^+^ANC visits and 4^+^ANC visits with individual, geographical and community-level explanatory variables using the prevalence measure, percentage change, and annual percentage change. We also examined the association between at least one and at least four antenatal care visits and independent variables using the *χ*^2^ test (since all the variables are categorical). To decompose the concentration index, explanatory variables were selected after the variable selection process in the logistic regression model in terms of the Akaike Information Criterion (AIC). The significant variables were included in the multivariable logistic regression model, and these variables contributed to the concentration index. The marginal effects, contribution to CI, and change of CI (%) were reported in this study. The Oaxaca-type decomposition and the Blinder-Oaxaca-type decomposition analysis were also performed. All the analyses were conducted by considering the survey weights using STATA 14.2 and R 4.1.1 software.

### Measuring and decomposing socio-economic inequality

#### Concentration index (CI)

Socio-economic inequalities in 1^+^ANC visits and 4^+^ANC visits were measured and investigated with the concentration index during the period 2011–2017. The concentration index is twice the area between the concentration curve and the equity line [40]. Antenatal care visits are more concentrated among the richest socioeconomic class when CI takes a positive value and the curve lies below the equity line. The concentration curve lies above the equity line when the ANC visits rate is higher in the poorest class. There is no socioeconomic inequality in ANC visits, when CI takes the value zero. The range of CI is -1 to +1 [41]. The covariance between ANC visits and proportional rank in wealth score is the concentration index [42] and is defined as follows:

$$CI=\frac{2}{n^{2}\bar{y}}\sum_{i=1}^{n}y_{i}r_{i}$$ (1)

Where, *y*_*i*_ and *r*_*i*_ are the variables of interest (1^+^ANC visits, 4^+^ANC visits) and fractional rank in the *i*^*th*^ individual according to wealth, respectively. For dichotomous variables of interest, CIs are not bounded between -1 and +1. To solve this problem, Wagstaff [43] and Erreyger [44] suggested different correction methods. Wagstaff normalized the CI in such a way that it is bounded between -1 to +1 and formalized as $CI_{Wag}=CI/1-\bar{y}$, where CI is defined in (1) and $\bar{y}$ is the mean of the interested health variable. In the case of the dichotomous outcome variable, Erreyger’s corrected concentration index can be defined as

$$ECI=\frac{4\times \bar{y}}{y^{max}-y^{min}}CI$$ (2)

Where *y*^*max*^ and *y*^*min*^ are the bounded values of *y*. The interpretation of ECI is similar to standard CI. Positive values of ECI indicate that the distribution of 1^+^ANC visits and 4^+^ANC visits favour economically affluent mothers.

#### Decomposition of CI

To measure the impact of important explanatory variables on the concentration index (for 1^+^ANC visits and 4^+^ANC visits) between 2011 and 2017, a decomposition analysis was conducted. The methods proposed by Wagstaff et al. [45] and Oaxaca [46] to decompose the socioeconomic inequality in 1^+^ANC visits and 4^+^ANC visits using regression analysis.

#### Wagstaff type decomposition

Consider the linear additive regression model that specifies the relationship between health outcome variable (*y*) and the set of *k* explanatory variables *x* and it can be defined as

$$y=\alpha +\sum_{k}\beta_{k}x_{k}+\varepsilon$$ (3)

According to Wagstaff type decomposition, the concentration index for outcome variables can be expressed as the weighted sum of the partial concentration indices for the independent factors of inequality, being weight the elasticity for each of the explanatory variables, *x*_*k*_:

$$CI=\sum_{k}\left(\frac{\beta_{k}{\bar{x}}_{k}}{\bar{y}}\right)CI_{k}+\frac{GCI_{e}}{\bar{y}}$$ (4)

In this study, both the dependent variables 1^+^ANC visits and 4^+^ANC visits are dichotomous. Consequently, the logistic regression model (non-linear model) was used to establish the relationship between outcome and explanatory variables[47]. Let *Y*_*k*_, (*k* = 1,2,…….,*n*), be a binary response variable (*Y* = 0/1) which follows Bernoulli distribution with the probability, *π*_*k*_ = Pr(*Y*_*k*_ = 1) and 1−*π*_*k*_ = Pr(*Y*_*k*_ = 0). Thus, the logistic regression model can be defined as $logit[\mathrm{Pr}\left(Y_{k}=1|x_{k}\right)]=log(\frac{\pi_{k}}{1-\pi_{k}})=\beta^{T}x_{k},$ where *β*^*T*^ is the regression coefficients vector of length (*k*+1), and *x* is the predictor matrix which has order *n*×(*k*+1). To conduct the decomposition analysis, some linear approximation is made to the logistic regression model [40]. The CI can be modified as:

$$CI=\sum_{k}\left(\frac{\beta_{k}^{m}{\bar{x}}_{k}}{\bar{y}}\right)CI_{k}+\frac{GCI_{e}}{\bar{y}}$$ (5)

Where, $\beta_{k}^{m}$ are the partial effects, ${\bar{x}}_{k}$ and *CI*_*k*_ are the mean and partial concentration indices of the explanatory variables, and *GCI*_*e*_ generalized concentration index of error. The decomposition of inequality can be expressed as follows (6) if we use Erreyger’s concentration index, however, it provides results as same as (5).


$$ECI=4.\sum_{k}(\beta_{k}^{m}{\bar{x}}_{k})ECI_{k}+GCI_{e}$$ (6)

#### Oaxaca type decomposition

In this study, we are also eager to know how socioeconomic inequality has changed during the period 2011–2017 (Δ*CI* = *CI*_*t*_−*CI*_*t*−1_) and the contribution of the associated factors to this change. We use the Oaxaca [45,46] type decomposition to decompose the ECI into the factors for this purpose. A factor contributes to the socioeconomic inequality in 1^+^ANC visits and 4^+^ANC visits if it is associated with outcome variables and asymmetrically distributed across socioeconomic groups that are measured by ECI [43,48]. The contribution of that variable is higher when it is more asymmetrically distributed and the partial effect is higher. The following Eqs (7) and (8) explain the variation of CI by changes in partial concentration index *CI*_*k*_ and elasticity $E_{t}={\beta_{k,t}{\bar{x}}_{k,t}/\bar{y}}_{t}$.


$$Variation1:\Delta CI=\sum_{k}E_{k,t-1}(CI_{k,t}-CI_{k,t-1})+\sum_{k}CI_{k,t}(E_{k,t}-E_{k,t-1})+\Delta \frac{GCI_{et}}{{\bar{y}}_{t}}$$ (7)

$$Variation2:\Delta CI=\sum_{k}{CI}_{k,t-1}(E_{k,t}-E_{k,t-1})+\sum_{k}E_{k,t}({CI}_{k,t}-{CI}_{k,t-1})+\Delta \frac{GCI_{et}}{{\bar{y}}_{t}}$$ (8)

Where, Δ indicates the difference across time. This method disentangled the inequality in 1^+^ANC and 4^+^ANC into two components. The first component in Eq (7) represents the changes of inequality in the factors and the second component assesses the changes in the susceptibility of the outcome variable concerning each independent variable over time [40].

#### Blinder-Oaxaca type decomposition

Although the Blinder-Oaxaca decomposition method was developed based on a multivariable linear regression model, Yun and Fairlie have extended the method for nonlinear models like the logistic regression model [28,49], which have been extensively used to quantify the contribution of factors to the socioeconomic inequality in health [29,50–52]. This decomposition method partitioned the group (white/black, urban/rural) differences into two components (i) explained difference is the compositional difference between groups and (ii) unexplained (coefficients) difference attributed due to the effect of the characteristics. In this paper, since the dependent variable is dichotomous, we used the decomposition method for a nonlinear model (logistic model) that has been given similar estimates of Fairlie decomposition. According to Fairlie, the decomposition for the 1^+^ANC (no/yes), 4^+^ANC (no/yes) differentials between urban and rural areas using the equation for logistic regression model of the following type, $P(Y=1)=F(X^{T}\hat{\beta })$ can be written as:

$${\bar{Y}}^{R}-{\bar{Y}}^{U}=\underset{Explained\;Part}{\underset{︸}{\left[\sum_{i=1}^{N^{R}}\frac{F(x_{i}^{R}{\hat{\beta }}_{R})}{N^{R}}-\sum_{i=1}^{N^{U}}\frac{F(x_{i}^{U}{\hat{\beta }}_{R})}{N^{U}}\right]}}+\underset{Unexplained\;Part}{\underset{︸}{\left[\sum_{i=1}^{N^{U}}\frac{F(x_{i}^{U}{\hat{\beta }}_{R})}{N^{U}}-\sum_{i=1}^{N^{R}}\frac{F(x_{i}^{U}{\hat{\beta }}_{U})}{N^{U}}\right]}}$$

Where *R* and *U* indicate rural and urban, ${\bar{Y}}^{m}$ and ${\bar{Y}}^{R}$ are the mean probabilities of 1^+^ANC (no/yes) or 4^+^ANC (no/yes) for a place of residence (urban/rural), $x_{i}^{R}$ and $x_{i}^{U}$ are the vectors of explanatory variables for urban and rural group, respectively. *F* represents the cumulative distribution function for logistic distribution, and *N* refers to the sample size for each group. An alternative equation to represent the non-linear decomposition is the following:

$${\bar{Y}}^{R}-{\bar{Y}}^{U}=\underset{Explained\;Part}{\underset{︸}{\left[\sum_{i=1}^{N^{R}}\frac{F(x_{i}^{R}{\hat{\beta }}_{U})}{N^{R}}-\sum_{i=1}^{N^{U}}\frac{F(x_{i}^{U}{\hat{\beta }}_{U})}{N^{U}}\right]}}+\underset{Unexplained\;Part}{\underset{︸}{\left[\sum_{i=1}^{N^{R}}\frac{F(x_{i}^{R}{\hat{\beta }}_{R})}{N^{R}}-\sum_{i=1}^{N^{R}}\frac{F(x_{i}^{R}{\hat{\beta }}_{U})}{N^{R}}\right]}}$$

In the Blinder-Oaxaca decomposition for the nonlinear model (Logistic regression), the "identification” and “path dependency" problems affect the estimated coefficients and inference. When we use nominal (categorical) variables as predictors, the choice of base category affects the decomposition estimates which are called the identification problem. To solve this problem, Yun suggested computing the normalized effects that are equivalent to taking the average of coefficient effects for a set of dummy variables when altering the reference groups. Path dependency is the sensitivity for ordering the variables included in the analysis. Fairlie proposed the solution to this issue of ordering the variables randomly across replications of the decomposition. This process needs an equal number of observations in both groups for one-to-one matching. Another solution is the selection of the random subsample from the larger group which is equal to the sample size of the smaller group and matches the response variable based on the predictive probability of each person. Individuals in each group were organized distinctly and matched based on ranking. Therefore, the contributions of each determinant are measured by matched observations to the outcome difference. Then, the mean estimate of subsamples (100/1000 times) is considered as the final estimate. Similar results were produced in both the Fairlie methods and nonlinear methods for disparity [29].

### Ethics statement

The authority of the Ministry of Health and Family Welfare, Bangladesh and the National Institute of Population Research and Training (NIPORT) implemented the 2004, 2007, 2011 and 2017 BDHS surveys. The ICF International of Calverton, Maryland, USA technically assisted Mitra and Associates, a Bangladeshi research firm located in Dhaka which conducted the surveys. The protocols of these surveys were approved by the National Research Ethics Committee of Bangladesh Medical Research Council (BMRC), Dhaka, Bangladesh and the Institutional Review Board of the InnerCity Fund (ICF) Macro, Maryland, USA. According to DHS ethics, verbal permission was obtained from all respondents before conducting the surveys. As per the standard protocol, the de-identified BDHS datasets were collected from the DHS website (https://dhsprogram.com/data/) by the mailed consent and taking an authentication letter from the authority for this study.

## Results

### Trends of 1^+^ANC and 4^+^ANC visits across factors

**Fig 1:**
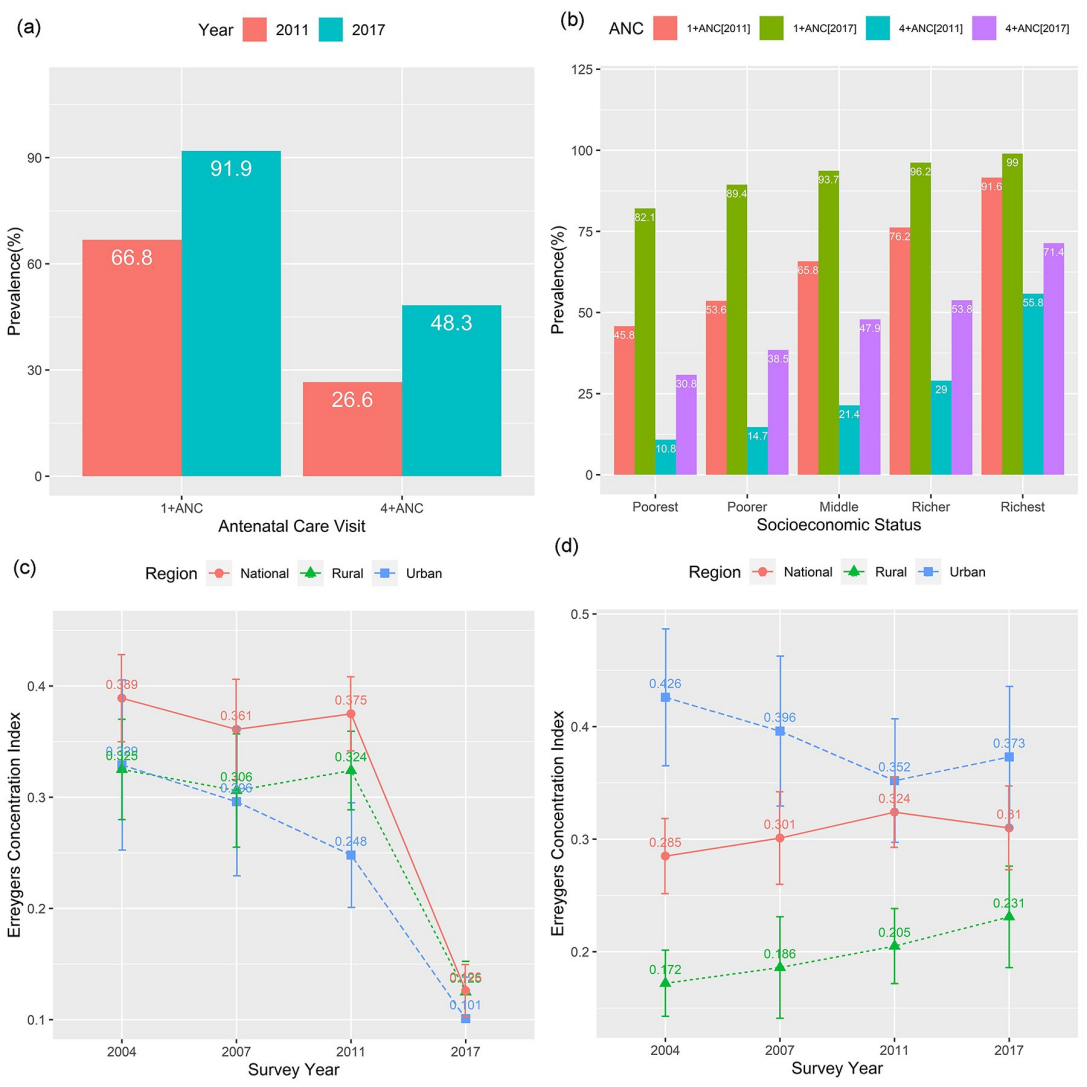
(a) Prevalence (%) of 1^+^ANC and 4^+^ANC for study periods (2011–2017) (b) Prevalence (%) of 1^+^ANC and 4^+^ANC across socioeconomic status for study periods (2011–2017) (c) Trends of socioeconomic inequality in 1^+^ANC across region from 2004 to 2017 (d) Trends of socioeconomic inequality in 4^+^ANC across region from 2004 to 2017.

We present the descriptive characteristics, bivariate analysis, and prevalence changes of the variables of interest (1^+^ANC, 4+ANC) in the study population over two survey years (Table 1, Fig 1(A) and 1(B)). After managing the data, 7178 mothers from 2011 and 4946 mothers from 2017 were included in our study. Between 2011 and 2017, the percentages of mothers who received at least one antenatal care (1^+^ANC) visit and at least four antenatal care 4^+^ANC visits increased from 66.8% to 91.9% and 26.6% to 48.3%, respectively (Fig 1(A)). However, the percentage gap between the poorest and richest is 82.1–99.0% and 30.8–71.4% for the respective variables (Fig 1(B)). We observed that the prevalence of 1^+^ANC and 4^+^ANC is lower among mothers with lower education, who live in Sylhet division, in rural areas, and have lower educated husbands. Similarly, lower prevalence rates were found among mothers who had watched TV not at all, wanted no more pregnancies, had birth order three, and were in the poorest group. After all, across all the determinants, both the 1^+^ANC and 4^+^ANC rates increased in 2017 compared to 2011 (Table 1).

**Table 1:** Prevalence trends of 1^+^ANC, and 4^+^ANC visits across the explanatory variables based on 2011 and 2017 BDHS data.

		At least one Antenatal Care Visit							4+ Antenatal Care Visits				
		2011		2017			Absolute Change (%)	Annual Change (%)	2011	2017		Absolute Change (%)	Annual Change (%)
		Size	Yes (%)	Size	Yes (%)	P- value			Yes (%)	Yes (%)	P- value		
Division***						<0.001					<0.001		
	Barisal	838	66.3	525	86.7		20.4	4.6	27.4	40.0		12.6	6.5
	Chittagong	1362	62.6	827	91.3		28.7	6.5	21.8	40.5		18.7	10.9
	Dhaka	1206	65.0	730	93.4		28.4	6.2	26.8	53.6		26.8	12.2
	Khulna	858	74.9	513	96.5		21.6	4.3	32.9	60.0		27.1	10.5
	Mymensingh			594	90.6					48.1			
	Rajshahi	935	72.1	520	95.4		23.3	4.8	24.9	50.4		25.5	12.5
	Rangpur	941	76.0	553	95.7		19.7	3.9	36.6	62.0		25.4	9.2
	Sylhet	1038	54.6	684	87.1		32.5	8.1	19.3	37.0		17.7	11.5
Place of Residence						<0.001					<0.001		
	Urban	2272	81.3	1699	94.9		13.6	2.6	43.5	59.3		15.8	5.3
	Rural	4906	60.0	3247	90.3		30.3	7.1	18.8	42.5		23.7	14.6
Women’s Education													
	No Education	1297	40.0	304	73.0		33.0	10.5	9.0	20.7		11.7	14.9
	Primary	2136	57.8	1370	86.9		29.1	7.0	16.6	34.2		17.6	12.8
	Secondary	3125	78.1	2372	94.6		16.5	3.2	32.5	51.5		19.0	8.0
	Higher	620	96.5	900	99.0		2.5	0.4	68.1	70.7		2.6	0.6
Last Birth C-Section						<0.001					<0.001		
	No	6015	61.9	3292	88.4		26.5	6.1	21.0	37.9		16.9	10.3
	Yes	1163	91.8	1654	98.9		7.1	1.2	55.4	68.9		13.5	3.7
Partner’s Education											<0.001		
	No Education	1917	47.6	692	82.5		34.9	9.6	11.8	30.1		18.3	16.9
	Primary	2084	62.6	1657	88.5		25.9	5.9	19.9	38.4		18.5	11.6
	Secondary	2156	76.3	1635	95.3		19.0	3.8	31.9	51.9		20.0	8.4
	Higher	1021	91.0	962	98.9		7.9	1.4	56.7	72.1		15.4	4.1
Partner’s Occupation						<0.001					<0.001		
	AGRWF	1904	52.6	981	86.7		34.1	8.7	14.1	36.5		22.4	17.2
	LDSOW	3014	68.3	2569	92.0		23.7	5.1	25.4	46.8		21.4	10.7
	PB	2085	77.6	1387	95.5		17.9	3.5	40.0	59.3		19.3	6.8
	UO	175	65.1	9	77.8		12.7	3.0	22.9	55.6		32.7	15.9
Wealth Status						<0.001					<0.001		
	Poorest	1486	45.8	1066	82.1		36.3	10.2	10.8	30.8		20.0	19.1
	Poorer	1372	53.6	998	89.4		35.8	8.9	14.7	38.5		23.8	17.4
	Middle	1384	65.8	891	93.7		27.9	6.1	21.4	47.9		26.5	14.4
	Richer	1444	76.2	980	96.2		20.0	4.0	29.0	53.8		24.8	10.8
	Richest	1492	91.6	1011	99.0		7.4	1.3	55.8	71.4		15.6	4.2
Watching TV						<0.001					<0.001		
	Not at all	2803	51.2	1884	85.2		34.0	8.9	13.7	34.0		20.3	16.4
	Less than once a week	870	63.7	434	92.4		28.7	6.4	20.3	45.4		25.1	14.4
	At least once a week	3505	79.9	2628	96.6		16.7	3.2	38.5	59.0		20.5	7.4
Birth Order Number						<0.001					<0.001		
	First	4557	74.6	3497	94.4		19.8	4.0	32.7	52.7		20.0	8.3
	Second	1945	58.5	1206	87.7		29.2	7.0	19.3	41.2		21.9	13.5
	Third	676	38.0	243	77.0		39.0	12.5	6.4	19.3		12.9	20.2
Pregnancy Wanted						<0.001					<0.001		
	Then	5082	69.4	3900	92.6		23.2	4.9	28.4	50.3		21.9	10.0
	Later	1129	68.1	643	92.1		24.0	5.2	28.3	46.5		18.2	8.6
	No more	967	51.3	403	85.4		34.1	8.9	15.0	31.8		16.8	13.3
Currently Working													
	No	6465	66.4	3098	93.0		26.6	5.8	26.4	48.7		22.3	10.7
	Yes	713	70.1	1848	90.2		20.1	4.3	28.1	47.6		19.5	9.2
	National	7178	66.8	4946	91.9		25.1	5.5	26.6	48.3		21.7	10.5

^a^AGRWF: Agriculture and Related Workers, Fisherman, LDSOW: Labour, Domestic Servent and other worker, PB: Professionals and Businessman, UO: Unemployed and Others.

The absolute changes in (4^+^ANC) prevalence over the period 2011 to 2017 was about 22% with an increased rate of 10.5% per annum (Table 1). Annual change (%) was comparatively higher in the Rajshahi division (12.5%), rural mothers (14.6%), uneducated mothers (14.9%), partners with no education (16.9%), and those in the poorest socio-economic class (19.1%). The rate of annual increase was 3.7% for C-section mothers and 10.3% for normal delivery mothers, respectively. We also found that the annual prevalence increase rate was higher among mothers who wanted no more pregnancies (13.3%), husbands with occupations related to agriculture (17.2%), those watching TV not at all (16.4%), and third babies (20.2%). For 1^+^ANC, the annual increase rate was 5.5%, while the absolute change was about 25% between the periods 2011–2017. Trends like as 4^+^ANC were found among all the determinants except division (Sylhet (8.1%)) in the context of absolute and annual changes of prevalence in ANC (Table 1).

The proportion of the explanatory variables by wealth status for two survey years, 2011, and 2017 are presented in S1 Table. We observed that about 19.4% of the poorest mothers lived in the Rangpur division and 23.1% of the richest mothers lived in the Dhaka division in 2011, whereas these figures were 19.1% and 27.7% in 2017. For both survey years, approximately 86% of the poorest mothers live in rural areas, while 69% of the richest mothers live in urban areas. In both survey years, the majority of the poorest mothers were in primary education (81%, 60%, respectively), while in the richest people, the percentage of secondary or higher education were 83%, and 89%, respectively. All other determinants can be described similarly. The proportion of 1+ANC and 4+ANC across wealth status is illustrated in Fig 1(B).

### Trends of wealth-based inequality in 1^+^ANC and 4^+^ANC visits

The wealth-based inequality for 1^+^ANC and 4^+^ANC visits in terms of CIs over the periods 2004 to 2017 are presented in Table 2, Fig 1(C) and 1(D). We observed that mothers from the richest households were more concentrated with 1^+^ANC and 4^+^ANC visits. The values of concentration indices indicated that overall inequalities in the 4^+^ANC were approximately parallel, while massive improvement was noticed in the 1^+^ANC, especially for 2017 (0.126) at the national level. Inequalities in 1^+^ANC among urban mothers gradually decreased (0.329 to 0.101), but the pattern for 4^+^ANC is inconsistent. Although, the CIs for 1^+^ANC showed an erratic trend for rural mothers until 2011, however, it declined to 0.125 in 2017. We also observed that wealth-based inequality in 4^+^ANC among rural mothers increased gradually from 0.172 to 0.231 from 2004 to 2017.

**Table 2:** Socioeconomic inequality in antenatal care visits and 4+ antenatal care visits from 2004–2017.

Concentration Index for ANC4					Concentration Index for ANC			
Year	ECI (SE)			H0: Diff = 0	ECI (SE)			H0: Diff = 0
	Urban	Rural	Overall	P-value	Urban	Rural	Overall	P-value
2004	0.426*(0.031)	0.172*(0.015)	0.285*(0.017)	0.00	0.329*(0.039)	0.325*(0.023)	0.389*(0.020)	0.32
2007	0.396*(0.034)	0.186*(0.023)	0.301*(0.021)	0.00	0.296*(0.034)	0.306*(0.026)	0.361*(0.023)	0.13
2011	0.352*(0.028)	0.205*(0.017)	0.324*(0.016)	0.00	0.248*(0.024)	0.324*(0.018)	0.375*(0.017)	0.00
2017	0.373*(0.032)	0.231*(0.023)	0.310*(0.019)	0.00	0.101*(0.019)	0.125*(0.014)	0.126*(0.012)	0.00

### Determinants of 1^+^ANC and 4^+^ANC Visits

The adjusted odds ratio with 95% confidence intervals and p-values that determine the significant odds ratios are shown in the multivariate logistic regression results (S2 Table). We found that mothers who lived in Rangpur, Khulna, Mymensingh and Rajshahi (OR: 2.64, 1.61, 1.39, and 1.18, respectively) divisions were significantly more likely to receive 4^+^ANC compared to the mothers who lived in Barisal division in 2017, whereas these results were opposite in 2011 except Rangpur division (OR: 1.89). The educational status of women and their husbands was positively associated with the 4^+^ANC. Higher-educated mothers have a 2.68 times higher chance of receiving 4^+^ANC than uneducated mothers in 2017 and a 3.85 times higher chance in 2011. For the husband’s educational status, these were 1.99 and 1.78 times higher. Rural mothers had a lower chance of receiving 4^+^ANC (OR: 0.74, and 0.53) than urban mothers in both survey periods. Women from comparatively wealthy households had a significantly higher chance of having 4^+^ANC than women from relatively poor households.

For instance, the likelihood of 4^+^ANC for the richest mothers was 2.36 times higher in 2017 and 2.84 times higher in 2011 than for the poorest mothers. Women who had their last birth by caesarean section (OR: 2.10, 2.03) and who watched TV frequently (OR: 1.38, 1.42) had a significantly higher chance of receiving 4^+^ANC for both periods. Birth order number (OR: 0.44, 0.64), pregnancy wanted (OR: 0.92, 0.67), and current working status (OR: 0.89, 1.22) were all negatively associated with 4^+^ANC in 2011 and 2017. Similar results were found for 1^+^ANC visits (yes/no) in both survey years, but the likelihood of receiving 1^+^ANC was higher than the likelihood of receiving 4^+^ANC for all determinants. Furthermore, when 1^+^ANC visits is the response variable, then the three predictor variables: a place of residence, pregnancy wanted, and current employment status were insignificant (p-value > 0.05) in the model.

### Determinants contributions to the inequality in 1^+^ANC and 4^+^ANC Visits

The effects and contributions of various determinants to the 1^+^ANC inequalities and 4^+^ANC inequalities, which are known as the decomposition analysis, are presented in Tables 3 and S3. Mainly, the tables show the effects of regression coefficients, predictor concentration index (CI), contribution and percentage contribution of the factors to the inequality of 1^+^ANC and 4^+^ANC. The positive values in the last column of the tables indicate that the predictor contribution to the CI increased in 2017 compared to 2011, while negative values indicate the opposite interpretation.

**Table 3:** Decomposition of Erreygers concentration index for 4^+^ antenatal care visits (Yes/No) 2011–2017.

		Marginal Effects & p-value				Marginal Effects *Mean		ECI		Contribution to CI		Contribution to CI (%)		
		2011		2017		2011	2017	2011	2017	2011	2017	2011	2017	Change
Division (Ref: Barisal)														
	Chittagong	-0.095	0.00	-0.054	0.11	-0.020	-0.012	0.060	0.071	-0.005	-0.003	-1.51	-1.05	0.46
	Dhaka	-0.070	0.01	0.003	0.93	-0.022	0.001	0.105	0.264	-0.009	0.001	-2.87	0.25	3.12
	Khulna	-0.041	0.12	0.099	0.01	-0.004	0.009	0.031	0.006	0.000	0.000	-0.15	0.07	0.22
	Mymensingh			0.068	0.09		0.006		-0.075		-0.002		-0.56	-0.56
	Rajshahi	-0.050	0.07	0.034	0.36	-0.007	0.004	-0.052	-0.036	0.001	-0.001	0.44	-0.18	-0.62
	Rangpur	0.109	0.00	0.198	0.00	0.012	0.021	-0.099	-0.136	-0.005	-0.011	-1.45	-3.68	-2.23
	Sylhet	-0.090	0.00	-0.005	0.89	-0.006	0.000	-0.008	-0.036	0.000	0.000	0.06	0.02	-0.04
Place of Residence (Ref: Urban)														
	Rural	-0.099	0.00	-0.062	0.01	-0.076	-0.045	-0.424	-0.412	0.128	0.075	39.59	24.09	-15.50
Women’s Education (Ref: No)														
	Primary	0.050	0.01	0.120	0.00	0.015	0.033	-0.213	-0.282	-0.013	-0.037	-3.95	-11.99	-8.04
	Secondary	0.098	0.00	0.173	0.00	0.042	0.085	0.330	0.078	0.056	0.027	17.26	8.55	-8.71
	Higher	0.206	0.00	0.201	0.00	0.016	0.035	0.181	0.286	0.011	0.039	3.49	12.72	9.23
Last Birth C-Section (Ref: No)														
	Yes	0.118	0.00	0.153	0.00	0.018	0.051	0.260	0.365	0.019	0.074	5.78	23.91	18.13
Partner’s Education (Ref: No)														
	Primary	0.012	0.42	0.006	0.81	0.004	0.002	-0.116	-0.275	-0.002	-0.002	-0.51	-0.73	-0.22
	Secondary	0.044	0.01	0.058	0.03	0.013	0.020	0.259	0.164	0.013	0.013	4.12	4.13	0.01
	Higher	0.086	0.00	0.147	0.00	0.011	0.027	0.270	0.312	0.012	0.034	3.76	10.92	7.15
Wealth Status (Ref: Poorest)														
	Poorer	0.011	0.57	0.009	0.72	0.002	0.002	-0.290	-0.313	-0.002	-0.002	-0.76	-0.70	0.06
	Middle	0.040	0.03	0.053	0.03	0.008	0.010	0.030	0.009	0.001	0.000	0.29	0.11	-0.18
	Richer	0.074	0.00	0.078	0.01	0.015	0.016	0.340	0.329	0.020	0.021	6.11	6.73	0.62
	Richest	0.162	0.00	0.184	0.00	0.030	0.036	0.604	0.630	0.073	0.091	22.48	29.32	6.84
Watching TV (Ref: Not at all)														
	Less than once a week	0.007	0.63	0.060	0.02	0.001	0.006	-0.092	-0.058	0.000	-0.001	-0.11	-0.41	-0.30
	At least once a week	0.046	0.00	0.073	0.00	0.022	0.040	0.645	0.565	0.056	0.089	17.38	28.83	11.45
Birth Order Number (Ref: First)														
	Second	-0.031	0.01	0.004	0.81	-0.008	0.001	-0.100	-0.097	0.003	0.000	1.04	-0.13	-1.17
	Third	-0.102	0.00	-0.092	0.04	-0.010	-0.004	-0.116	-0.060	0.004	0.001	1.39	0.35	-1.04
Pregnancy Wanted (Ref: Then)														
	Later	0.001	0.90	-0.057	0.01	0.000	-0.007	0.000	-0.012	0.000	0.000	0.00	0.12	0.12
	No more	-0.012	0.51	-0.082	0.00	-0.002	-0.007	-0.107	-0.049	0.001	0.001	0.23	0.41	0.19
Currently Working (Ref: No)														
	Yes	-0.017	0.31	0.040	0.02	-0.002	0.015	0.020	-0.254	0.000	-0.015	-0.04	-4.88	-4.84
	Explained									0.363	0.391	112.05	126.22	14.17
	Unexplained									-0.039	-0.081	-12.05	-26.22	
	Total									0.324	0.310	100	100	

**Fig 2:**
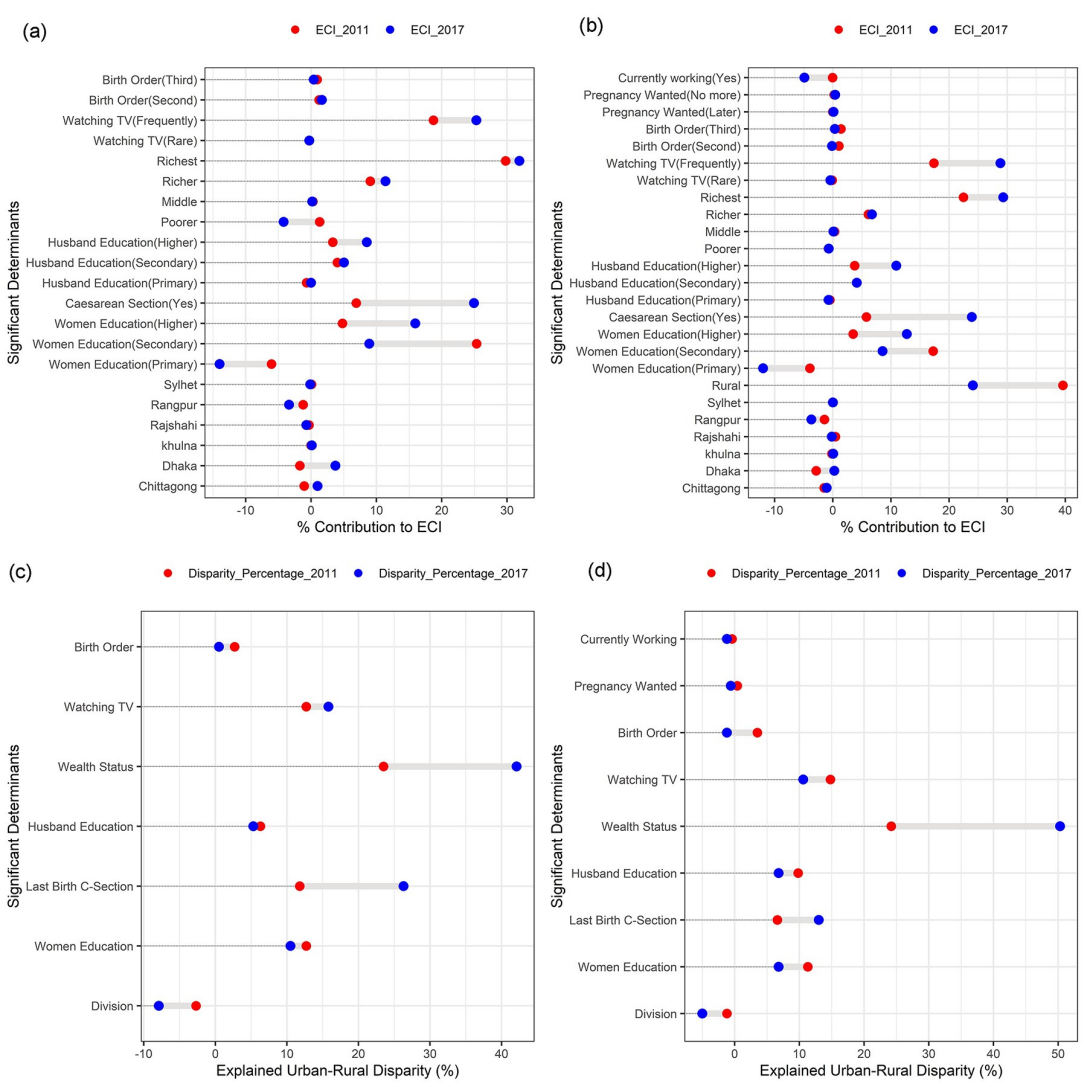
(a) Contribution (%) changes of determinants to the ECI in 1^+^ANC from 2011 to 2017 (b)) Contribution (%) changes of determinants to the ECI in 4^+^ANC from 2011 to 2017 (c) Urban-rural disparity (%) changes of determinants for 1^+^ANC from 2011 to 2017 (d) Urban-rural disparity (%) changes of determinants for 4^+^ANC from 2011 to 2017.

From Tables 3 and S3, we observe that respondent place of residence accounts for about 39.6% of the wealth-based inequality in 4^+^ANC in 2011 and 24.1% in 2017, whereas this factor has a statistically insignificant contribution to inequality in 1^+^ANC. The contribution of women’s education reduced from 16.8% in 2011 to 9.28% in 2017 for 4^+^ANC and from 24.1% to 10.87% for 1^+^ANC. On the other hand, husband education was responsible for 14.3% and 13.6% of inequality in 2017 for 4^+^ANC and 1^+^ANC, respectively, compared to 7.4% and 6.7% in 2011. We also found that the mother’s last delivery (Caesarean section) had a relatively greater contribution to inequality in 2017 than in 2011 for both 1^+^ANC (5.78–23.9%) and 4^+^ANC (6.9–24.9%). Among the factors, household socioeconomic status has a significant impact on inequality in 1^+^ANC (2011: 40.5%, 2017: 39.3%) and 4^+^ANC (2011: 28.1%, 2017: 35.5%). Watching television explained 17.3% of the inequality in 4^+^ANC in 2011, which increased to 28.4% in 2017, and it was also one of the most significant contributors to 1^+^ANC inequality in both years (2011: 18.5%, 2017: 25.0%). Although, the predictors: birth order, pregnancy wanted, and current working status were significant in the regression model for 4^+^ANC, they had a very small contribution to the decline or upturn in inequality. Moreover, the variables pregnancy wanted and current working status were insignificant in the model for 1^+^ANC. The changes in contribution for different factors are illustrated in Fig 2(A) and 2(B).

### Decomposition of changes in inequality in 1^+^ANC and 4^+^ANC visits

Table 4, Fig 2(A) and 2(B), shows the results of the Oaxaca-type decomposition that decomposes the changes in inequality over the period 2011 to 2017. Mother’s place of residence was the most important factor in changes (52.1%) in the inequality in 4+ANC, but its effect on the inequality in 1+ANC was insignificant. Approximately 23% (sum of changes (%) for women’s education) change in inequality was observed in 4+ANC due to women’s education; whereas it was approximately 21% in 1+ANC. Secondary education has more impact on positive changes (24.5% and 22.4%, respectively). The collective change in inequality in 1^+^ANC was about 30% for the cause of wealth status and about 20% for 4^+^ANC, particularly the sensitivity effects of the richest portion were larger positive contributions (15.3% and 21.4%). The geographical variable, division, was negatively associated with changes in inequality for both of the targeted variables. Caesarean section changed the inequality by -11% for 4^+^ANC, but for 1^+^ANC it has a small contribution. Mothers who watch TV frequently had a greater influence to change the inequality in a positive direction for 4^+^ANC (6.2%) and 1^+^ANC (12.3%). The changes in inequality in 4^+^ANC were also associated with the factors of birth order (4.3%) and current working status (4.4%).

**Table 4:** Oaxaca type decomposition for changes in inequality for antenatal care visits 2011–2017.

		4+ Antenatal Care Visits (≥4 = 1, <4 = 0)						Antenatal Care Visits (Yes/No)					
		Variation (1)		Variation (2)		Total	%	Variation (1)		Variation (2)		Total	%
Division (Ref: Barishal)													
	Chittagong	0.000	0.004	-0.001	0.004	0.003	-1.87	0.000	0.0018	-0.0003	0.0021	0.0018	-1.23
	Dhaka	0.000	0.010	-0.015	0.025	0.010	-5.60	0.001	0.0030	-0.0038	0.0075	0.0038	-2.50
	Khulna	0.000	0.001	0.000	0.000	0.001	-0.34	-0.0002	0.0002	2.3E-07	3.6E-05	0.0000	-0.02
	Mymensingh	-0.001	0.000	0.000	-0.001	-0.001	0.51	-0.0002	0.0000	0.0000	-0.0002	-0.0002	0.15
	Rajshahi	0.000	-0.002	0.000	-0.001	-0.002	0.99	0.0001	0.0001	0.0001	7.6E-05	0.0002	-0.15
	Rangpur	-0.002	0.001	-0.002	0.001	-0.001	0.64	-0.0003	0.0009	-0.0007	0.0013	0.0006	-0.40
	Sylhet	0.000	0.000	0.001	-0.001	0.000	0.10	0.0000	-0.0001	0.0003	-0.0005	-0.0001	0.09
Place of Residence (Ref: Urban)													
	Rural	-0.001	-0.093	-0.004	-0.090	-0.094	52.09						
Women’s Education (Ref: No)													
	Primary	-0.005	-0.002	-0.004	-0.002	-0.006	3.54	-0.0012	0.0051	-0.0029	0.0068	0.0040	-2.63
	Secondary	-0.045	0.001	-0.044	0.000	-0.044	24.49	-0.0098	-0.0238	-0.0280	-0.0056	-0.0337	22.4
	Higher	0.008	0.001	0.007	0.002	0.009	-5.07	0.0020	-0.0035	0.0041	-0.0055	-0.0015	0.99
Last Birth C-Section (Ref: No)													
	Yes	0.011	0.009	0.008	0.012	0.020	-11.00	0.0025	-0.0039	0.0041	-0.0055	-0.0015	0.98
Partner’s Education (Ref: No)													
	Primary	-0.001	0.001	-0.002	0.003	0.001	-0.30	-8.8E-07	0.0010	-0.0014	0.0023	0.0010	-0.65
	Secondary	-0.004	-0.003	-0.005	-0.002	-0.007	3.94	-0.0010	-0.0031	-0.0021	-0.0020	-0.0041	2.72
	Higher	0.002	0.003	0.002	0.003	0.005	-2.92	0.0004	-0.0023	0.0008	-0.0026	-0.0019	1.26
Wealth Status (Ref: Poorest)													
	Poorer	0.000	0.002	0.000	0.002	0.001	-0.79	-0.0001	-0.0032	0.0002	-0.0035	-0.0034	2.23
	Middle	0.000	0.000	-0.001	0.000	-0.001	0.45	-0.0002	-0.0002	-0.0003	-5.7E-05	-0.0004	0.24
	Richer	0.000	-0.009	-0.001	-0.009	-0.010	5.29	-0.0001	-0.0091	-0.0004	-0.0088	-0.0092	6.13
	Richest	0.002	-0.030	0.003	-0.031	-0.028	15.31	0.0004	-0.0326	0.0018	-0.0340	-0.0322	21.4
Watching TV (Ref: Not at all)													
	Less than once a week	0.000	-0.001	0.000	0.000	0.000	0.17	0.0001	0.0002	0.0001	0.0001	0.0003	-0.18
	At least once a week	-0.007	-0.004	-0.007	-0.004	-0.011	6.22	-0.0012	-0.0172	-0.0034	-0.0151	-0.0185	12.3
Birth Order Number (Ref: First)													
	Second	0.000	-0.004	0.000	-0.004	-0.004	2.06	-1.8E-05	-0.0012	-5.3E-05	-0.0012	-0.0012	0.80
	Third	-0.001	-0.004	-0.002	-0.002	-0.004	2.27	-0.0001	-0.0011	-0.0007	-0.0006	-0.0012	0.82
Pregnancy Wanted (Ref: Then)													
	Later	0.000	0.000	0.000	0.000	0.000	-0.11						
	No more	-0.001	0.001	0.000	0.000	0.000	0.05						
Currently Working (Ref: No)													
	Yes	-0.009	0.001	0.002	-0.010	-0.008	4.38						
	Unexplained					-0.010	5.49					-0.0531	35.30
	Total					-0.180	99.99					-0.1504	100.00

### Trend and decomposition of urban-rural disparity in 1^+^ANC and 4^+^ANC visits

The decomposition results of the predicted urban-rural difference in 1^+^ANC and 4^+^ANC using the Blinder-Oaxaca decomposition for the logistic regression model are presented in Table 5, Fig 2(B) and 2(C). In our analysis, the predicted probability of 4^+^ANC was 0.437 for urban mothers and 0.181 for rural mothers, yielding in a 4^+^ANC disparity of 0.256 in 2011, and the disparity was (0.589–0.429) 0.161 in 2017. Consequently, urban and rural mothers differed by 25.6 and 16.1 percentage points in 2011 and 2017, respectively. Negative values of the estimated coefficient indicate that they have opposite effects in explaining the disparity. As shown in Table 5, about 69% of the total urban-rural disparities were explained by the difference in the level of observed factors in 2011 and it was 79.5% in 2017. A major portion of the disparity was explained by household wealth status. The contribution was 24.2% in 2011 and 50.3% in 2017. These findings indicate that if a higher proportion of rural mothers were the richest, the likelihood of receiving 4^+^ANC has increased by 24.2% in 2011 and 50.3% in 2017, reducing the disparity. Women’s education and husbands education explain the disparity of 11.3%, 9.8% in 2011 and 6.8%, 6.8% in 2017, respectively. The disparity explained by mothers whose last birth was by caesarean section has increased from 6.6% in 2011 to 13% in 2017. This is an indicator of increased caesarean section among urban mothers, and they are more interested in receiving health services. Due to watching television, disparity decreased from 14.8% to 10.6% in 2017, representing a reduction in disparity as rural mother awareness increased. Regional variation has a reverse impact on explaining the urban-rural disparity in 4^+^ANC. Similarly, we can interpret the results of urban-rural disparity in the case of 1^+^ANC visits. Notably, the disparity for 1^+^ANC dramatically declined in 2017 (0.036), and the disparity for 4^+^ANC was bigger than the disparity for 1^+^ANC for the two study periods. Finally, although the urban-rural gap has reduced to receive 1^+^ANC, it remains at an alarming level of 4^+^ANC.

**Table 5:** Two-fold Blinder-Oaxaca decomposition of urban-rural disparity in 4^+^ANC and 1^+^ANC into the significant predictors.

	4+ Antenatal Care Visits ((≥4 = 1, <4 = 0)				Antenatal Care Visits (Yes/No)			
	2011		2017		2011		2017	
	Coefficients	Explained (%)	Coefficients	Explained (%)	Coefficients	Explained (%)	Coefficients	Explained (%)
Urban Mothers	0.437		0.589		0.817		0.948	
Rural Mothers	0.181		0.428		0.596		0.910	
Difference	0.256		0.161		0.221		0.038	
Explained	0.177	69.2	0.128	79.5	0.147	66.5	0.036	94.7
Unexplained	0.079	30.8	0.034	21.1	0.075	33.9	0.002	5.3
Disparity explained by predictors					Disparity explained by predictors			
Division	-0.003	-1.2	-0.008	-5.0	-0.006	-2.7	-0.003	-7.9
Women’s Education	0.029	11.3	0.011	6.8	0.028	12.7	0.004	10.5
Last Birth C-Section	0.017	6.6	0.021	13.0	0.026	11.8	0.010	26.3
Partner’s Education	0.025	9.8	0.011	6.8	0.014	6.3	0.002	5.3
Wealth Status	0.062	24.2	0.081	50.3	0.052	23.5	0.016	42.1
Watching TV	0.038	14.8	0.017	10.6	0.028	12.7	0.006	15.8
Birth Order Number	0.009	3.5	-0.002	-1.2	0.006	2.7	0.0002	0.5
Pregnancy Wanted	0.001	0.4	-0.001	-0.6				
Currently Working	-0.001	-0.4	-0.002	-1.2				
Total		100		100.6		100.4		100

## Discussion

Over the past two decades, Bangladesh has made significant progress in improving the health of children and mothers [53,54]. However, the wealth-based inequality in maternal health care services like antenatal care visits remains a major concern in Bangladesh[14,55]. This study explored and examined the trend of 1+ANC and 4+ANC based on prevalence (%) and wealth-based inequality in Bangladesh between 2011 and 2017. We have identified the potential factors and their contributions to the inequalities in 1+ANC and 4+ANC at the national level, along with the factors that contribute to the urban-rural disparity. In our analysis, we also conducted comparisons in three dimensions, including over different periods, urban-rural areas, 1+ANC, and 4+ANC.

The rates of both 1+ANC and WHO-recommended 4+ANC were on an upward trend in Bangladesh over the specified periods, even though the rate of4+ANC visits remained below 50%. Similar rates of 4+ANC visits have also been observed in other developing countries [56,57]. In this particular context, Bangladesh has made a policy to ensure that pregnant women receive at least 4+ANC visits, with the first ANC visit occurring before the completion of 4 months of gestational age [14]. The results of bivariate and logistic regression analysis showed that the educated mothers and educated husbands, place of residence, last birth caesarean section, socioeconomic status, division, watching TV, and birth order were more important factors in changing the prevalence of 1+ANC and 4+ANC visits. Our findings have been supported by numerous investigations conducted in Bangladesh and other locations over the past decade [13,14,55–58]. The disparity between the poorest and richest individuals poses a significant challenge in ensuring equal access to antenatal care services for all mothers. Our study found significant socioeconomic inequality in the utilization of 1+ANC and 4+ANC services between 2004 and 2017, which is consistent with previous studies on socioeconomic inequality [13,14,35]. The concentration index analysis reveals that the inequities in 4+ANC exhibited inconsistency at the national and urban levels from 2004 to 2017, while it consistently increased in rural Bangladesh. However, there was a slight increase at the national level and a minor decline in urban Bangladesh. Conversely, the inequities in 1+ANC at the national and rural levels had a comparable trajectory until 2011 but had substantially decreased by 2017, although being steadily reduced over the periods in urban Bangladesh.

The Oaxaca-type decomposition analysis showed and explained the contributing factors to the changes in the observed inequality. We found that 1^+^ANC and 4^+^ANC were more concentrated among the advantaged mothers with high levels of socioeconomic status and educated mothers than others, which is compatible with the results of similar studies in different developing countries [59–62]. This result indicates the significant association of these variables with the pro-rich inequality. Richer women were more interested in paying for receiving the 1^+^ANC and 4^+^ANC and were more aware of health communication, health services and medical complications, while poorer women were unable to bear transportation costs to receive publicly available health facilities [45,60]. The outcome of the decomposition analysis also clarifies that women who were married to educated husbands played a constructive role in the changes in observed inequality. Among the various factors influencing pro-rich inequality in 4^+^ANC, women’s place of residence emerges as one of the most significant contributors. However, this factor holds no significance in the case of 1^+^ANC, indicating that rural women have contributed to the rise in inequality. In most developing countries, poorer women in rural areas are more concentrated, but they have limited access to healthcare services such as 4^+^ANC [60]. Moreover, watching TV played a significant role in elucidating the wealth-based inequality in both 1^+^ANC and 4^+^ANC. Mothers who prioritized watching TV had a significant impact on changes in socioeconomic inequality due to their awareness of health facilities and utilization of services. Another influential factor in the changes of inequality for both variables 1^+^ANC and 4^+^ANC was the last birth, which involved a caesarean section. Caesarean-section mothers were attentive to their healthcare due to their previous health status (caesarean delivery). Though some predictors such as divisional region, birth order, current working status, and pregnancy wanted were significant in the model, they had less impact on changes in pro-rich inequality.

The Blinder Oaxaca type decomposition method describes and elucidates the disparity in the utilization of 1^+^ANC and 4^+^ANC services between urban and rural women. The disparity in taking 4+ANC was greater in the case of 1+ANC, whereas there was a decrease in both response variables in 2017 as compared to 2011. Wealth status was the most dominant predictor in explaining the urban-rural disparity for all scenarios like as the Oaxaca type decomposition. The disparity can be significantly explained by educated women who married an educated husband with a higher socioeconomic status, as these women were less prevalent in rural areas compared to urban areas [10]. Urban women watched TV more frequently than rural women which increased the disparity [63]. Like as the Oaxaca-type decomposition, last birth by caesarean section was another leading variable that changed the gap between urban and rural women in the case of 1^+^ANC and 4^+^ANC. This was due to a larger proportion of caesarean women living in urban areas [64]. The predicted difference between the urban and rural women to accept the services of 1^+^ANC and 4^+^ANC was explained negatively by the respondents’ division.

## Limitations

There are certain constraints in our study. For instance, we measured the trend of 1^+^ANC and 4^+^ANC through cross-sectional data, yet the impact of 1^+^ANC and 4^+^ANC rates could not be observed without longitudinal data. Supply-side factors were excluded from the models when assessing the wealth-based inequality in 1^+^ANC and 4^+^ANC. Additionally, recall and social factor biases occurred due to the use of self-reported BDHS data, which may have influenced our study findings. However, childbirth is a very crucial event for every mother, and only the latest birth may alleviate this limitation [12]. Another limitation is that decomposition analysis accounts for the factors that contribute to the inequality but it does not provide any causal inference [65]. Finally, any causal inference was not possible on our findings due to the data that were taken from a cross-sectional study.

## Conclusions and recommendations

The study findings indicate that, although the prevalence of 1^+^ANC and 4^+^ANC is increasing over the study periods and socioeconomic inequality is declining, it continues to persist. Hence, policymakers should implement community-based interventions or policies that target potential factors for improving maternal health. Based on the results, our study has generated some useful recommendations. Over the last two decades, Bangladesh has taken different policies and programs to provide equal access to health care for rich and poor mothers to reduce child and maternal mortality, and these policies have had an impact on the 1^+^ANC and 4^+^ANC rates [8,10,63]. Although socioeconomic inequality for 1^+^ANC decreased significantly in 2017, there was no progress observed for 4^+^ANC. Therefore, policymakers should prioritize making maternal health care services more accessible, particularly for antenatal care visits for low-income women. Given the findings of our research, it is evident that both women’s education and their husbands’ education play a crucial role in describing the inequality in 4^+^ANC, the government should emphasise policies that provide on delivering high-quality education and ensure the successful completion of education for women, especially for rural women. The income gap has been expanding recently along with Bangladesh’s economic growth. In this regard, the government and policymakers should undertake programs to reduce income disparities, while our findings reflect that wealth status has a greater impact on inequality. The role of frequently watching TV has a large involvement in inequality. To address this issue, it is crucial to launch different intervention programs that aim to enhance awareness among women. Furthermore, socioeconomic inequality is influenced by factors such as regional variation, birth order, and the method of delivery for the last birth, specifically caesarean section. Finally, the government should special focus on rural women to bring them into the mainstream of receiving antenatal care, since our study highlights a significant disparity between urban and rural women for the study periods.

## Supporting information

S1 Fig (a) Contribution (%) of determinants to the ECI in 1+ANC for 2011 (b) Contribution (%) of determinants to the ECI in 4+ANC for 2011 (c) Contribution (%) of determinants to the ECI in 1+ANC for 2017 (d) Contribution (%) of determinants to the ECI in 4+ANC for 2017.
(TIF)

S2 Fig (a) Contribution (%) of determinants to the urban-rural disparity in 1+ANC for 2011 (b) Contribution (%) of determinants to the urban-rural disparity in 4+ANC for 2011 (c) Contribution (%) of determinants to the urban-rural disparity in 1+ANC for 2017 (d) Contribution (%) of determinants to the urban-rural disparity in 4+ANC for 2017.
(TIF)

S1 Table Distribution of the explanatory variables by wealth status based on 2011 and 2017 BDHS data. (DOCX)

S2 Table Logistic regression results for the factors associated with 4^+^antenatal care visits and 1^+^antenatal care visits. (DOCX)

S3 Table Decomposition of Erreygers concentration index for 1^+^antenatal care visits (Yes/No), 2011–2017. (DOCX)
