# Supplementary figures and images for: Socioeconomic inequality and urban-rural disparity of antenatal care visits in Bangladesh: A trend and decomposition analysis
Source: PLoS One. 2024 Mar 25;19(3):e0301106. doi: 10.1371/journal.pone.0301106 (PMC10962795; doi:10.1371/journal.pone.0301106)

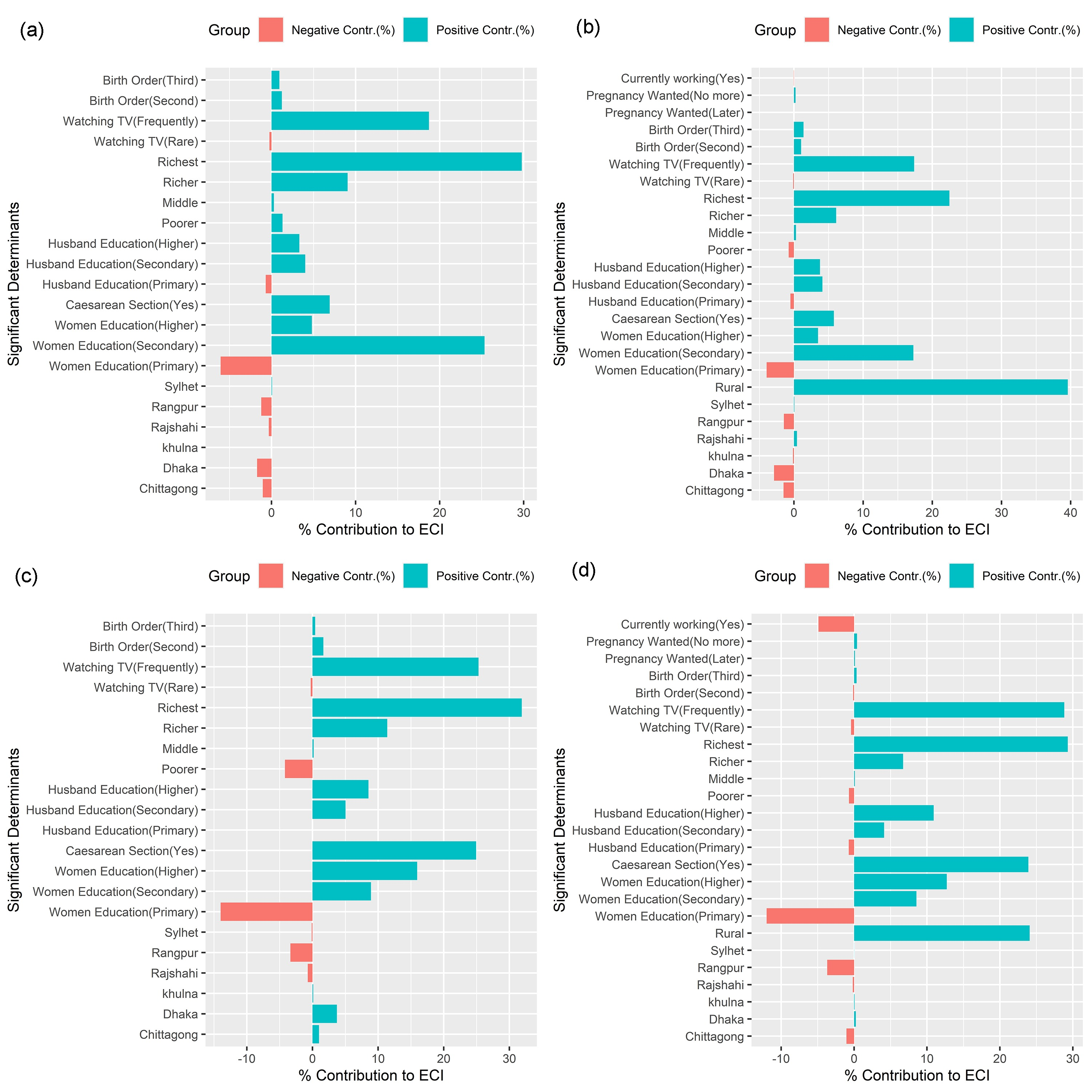

Supplement: S1 Fig — (a) Contribution (%) of determinants to the ECI in 1+ANC for 2011 (b) Contribution (%) of determinants to the ECI in 4+ANC for 2011 (c) Contribution (%) of determinants to the ECI in 1+ANC for 2017 (d) Contribution (%) of determinants to the ECI in 4+ANC for 2017. (TIF) [file pone.0301106.s001.tif]

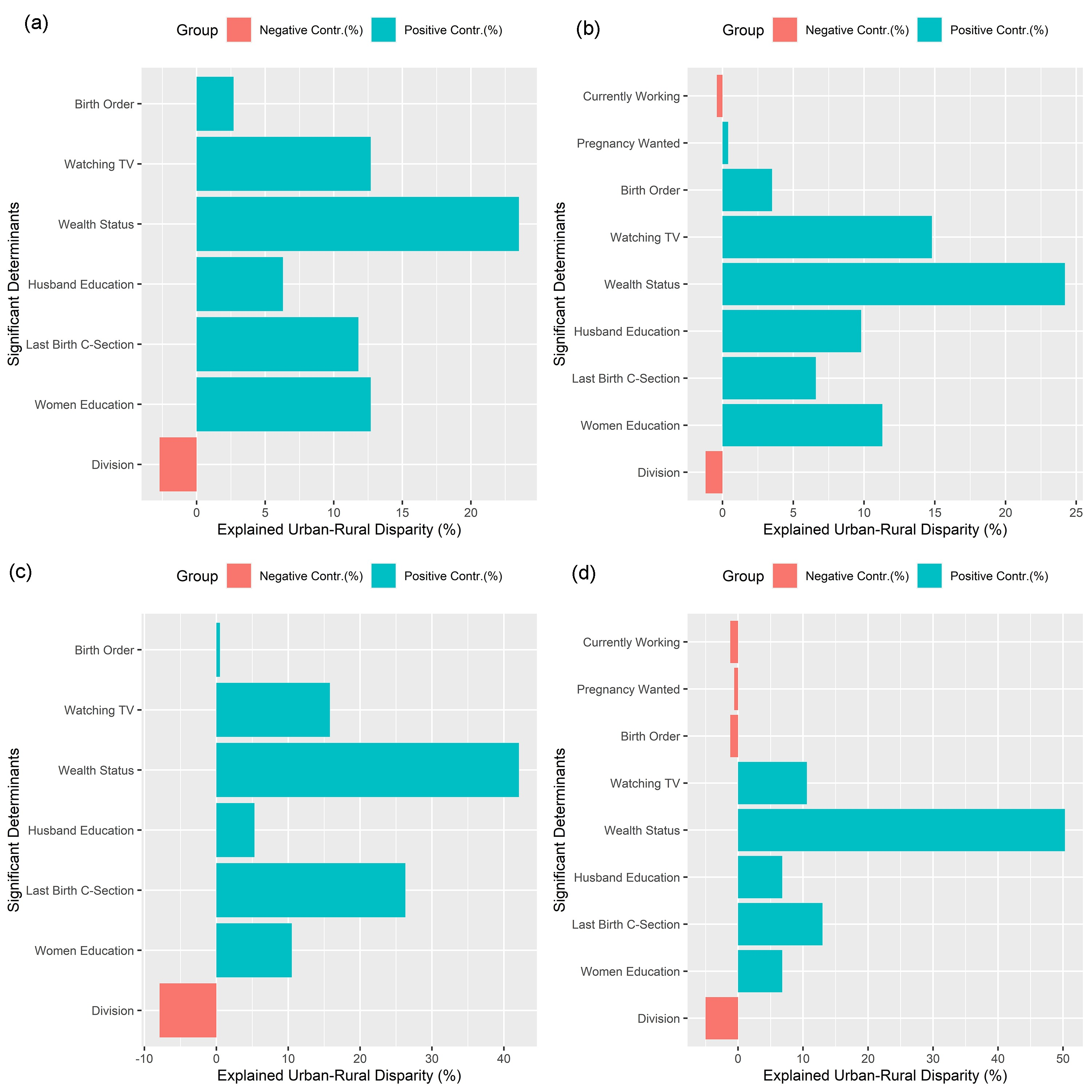

Supplement: S2 Fig — (a) Contribution (%) of determinants to the urban-rural disparity in 1+ANC for 2011 (b) Contribution (%) of determinants to the urban-rural disparity in 4+ANC for 2011 (c) Contribution (%) of determinants to the urban-rural disparity in 1+ANC for 2017 (d) Contribution (%) of determinants to the urban-rural disparity in 4+ANC for 2017. (TIF) [file pone.0301106.s002.tif]
