# Supplementary material for: Socioeconomic inequality and urban-rural disparity of antenatal care visits in Bangladesh: A trend and decomposition analysis
Source: PLoS One. 2024 Mar 25;19(3):e0301106. doi: 10.1371/journal.pone.0301106 (PMC10962795; doi:10.1371/journal.pone.0301106)
Supplement: S1 Table — (DOCX) [file pone.0301106.s003.docx]

S1 Table. Distribution of the explanatory variables by wealth status based on 2011 and 2017 BDHS data.

|  | | Wealth Index Quintiles (%) | | | | | | | | | |
| --- | --- | --- | --- | --- | --- | --- | --- | --- | --- | --- | --- |
|  | | 2011 | | | | | 2017 | | | | |
|  | | Poorest | Poorer | Middle | Richer | Richest | Poorest | Poorer | Middle | Richer | Richest |
| At least one ANC | |  |  |  |  |  |  |  |  |  |  |
|  | No | 54.2 | 46.4 | 34.2 | 23.8 | 8.4 | 17.9 | 10.6 | 6.3 | 3.8 | 1.0 |
|  | Yes | 45.8 | 53.6 | 65.8 | 76.2 | 91.6 | 82.1 | 89.4 | 93.7 | 96.2 | 99 |
| At least four ANC | |  |  |  |  |  |  |  |  |  |  |
|  | No | 89.2 | 85.3 | 78.6 | 71.0 | 44.2 | 69.2 | 61.5 | 52.1 | 46.2 | 28.6 |
|  | Yes | 10.8 | 14.7 | 21.4 | 29.0 | 55.8 | 30.8 | 38.5 | 47.9 | 53.8 | 71.4 |
| Division*** | |  |  |  |  |  |  |  |  |  |  |
|  | Barisal | 12.6 | 15.8 | 13.3 | 10.6 | 6.5 | 15.5 | 12.0 | 11.8 | 8.7 | 4.9 |
|  | Chittagong | 14.7 | 16.5 | 18.3 | 24.2 | 21.0 | 13.2 | 11.9 | 18.9 | 18.3 | 21.8 |
|  | Dhaka | 16.2 | 13.8 | 14.2 | 16.3 | 23.0 | 5.5 | 8.4 | 12.7 | 19.8 | 27.7 |
|  | Khulna | 8.6 | 10.3 | 13.9 | 13.5 | 13.5 | 6.1 | 10.5 | 13.2 | 12.1 | 10.5 |
|  | Mymensingh |  |  |  |  |  | 15.7 | 17.2 | 11.6 | 9.3 | 6.0 |
|  | Rajshahi | 12.5 | 14.1 | 15.6 | 13.1 | 10.1 | 8.5 | 12.7 | 12.5 | 11.8 | 7.4 |
|  | Rangpur | 19.4 | 17.4 | 12.0 | 9.8 | 7.1 | 19.1 | 13.1 | 9.3 | 6.6 | 6.9 |
|  | Sylhet | 16.0 | 12.0 | 12.7 | 12.4 | 18.8 | 16.3 | 14.0 | 10.1 | 13.4 | 14.7 |
| Place of Residence | |  |  |  |  |  |  |  |  |  |  |
|  | Urban | 13.7 | 12.2 | 20.2 | 40.7 | 69.2 | 17.4 | 14.0 | 25.9 | 45.1 | 69.3 |
|  | Rural | 86.3 | 87.8 | 79.8 | 59.3 | 30.8 | 82.6 | 86.0 | 74.1 | 54.9 | 30.7 |
| Women’s Education | |  |  |  |  |  |  |  |  |  |  |
|  | No Education | 41.1 | 22.4 | 12.2 | 9.8 | 4.5 | 13.5 | 6.5 | 5.2 | 3.3 | 1.7 |
|  | Primary | 39.9 | 38.9 | 32.4 | 25.7 | 12.7 | 46.2 | 35.1 | 25.4 | 21.3 | 9.1 |
|  | Secondary | 18.7 | 37.6 | 50.7 | 55.3 | 55.7 | 36.7 | 51.1 | 55.3 | 53.7 | 44.7 |
|  | Higher | 0.3 | 1.0 | 4.6 | 9.2 | 27.1 | 3.6 | 7.3 | 14.1 | 21.7 | 44.5 |
| Last Birth C-Section | |  |  |  |  |  |  |  |  |  |  |
|  | No | 96.9 | 93.2 | 88.2 | 82.2 | 59.5 | 87.2 | 77.9 | 68.2 | 61.9 | 36.6 |
|  | Yes | 3.1 | 6.8 | 11.8 | 17.8 | 40.5 | 12.8 | 22.1 | 31.8 | 38.1 | 63.4 |
| Partner’s Education | |  |  |  |  |  |  |  |  |  |  |
|  | No Education | 56.0 | 35.9 | 22.1 | 14.2 | 5.4 | 28.2 | 18.2 | 11.9 | 7.6 | 2.9 |
|  | Primary | 33.0 | 36.2 | 32.2 | 29.4 | 15.2 | 47.9 | 45.0 | 34.3 | 27.0 | 12.5 |
|  | Secondary | 10.5 | 24.0 | 36.8 | 39.5 | 39.6 | 19.9 | 29.7 | 38.6 | 43.7 | 35.1 |
|  | Higher | 0.5 | 3.9 | 8.9 | 16.9 | 39.7 | 3.9 | 7.1 | 15.2 | 21.7 | 49.6 |
| Partner’s Occupation | |  |  |  |  |  |  |  |  |  |  |
|  | AGRWF | 45.6 | 39.9 | 27.2 | 15.9 | 4.8 | 35.0 | 27.5 | 20.2 | 11.3 | 4.3 |
|  | LDSOW | 43.5 | 41.3 | 44.4 | 46.2 | 34.8 | 52.4 | 52.8 | 54.7 | 57.8 | 42.5 |
|  | PB | 19.3 | 17.0 | 25.6 | 35.2 | 57.0 | 12.5 | 19.4 | 24.8 | 30.8 | 53.1 |
|  | UO | 1.5 | 1.7 | 2.9 | 2.6 | 3.4 | 0.1 | 0.3 | 0.3 | 0.1 | 0.1 |
| Watching TV | |  |  |  |  |  |  |  |  |  |  |
|  | Not at all | 72.2 | 59.5 | 38.6 | 19.7 | 6.3 | 77.3 | 46.5 | 31.8 | 22.2 | 9.4 |
|  | Less than once a week | 14..5 | 17.3 | 15.6 | 9.6 | 4.2 | 8.9 | 14.2 | 8.4 | 7.3 | 54.9 |
|  | At least once a week | 13.3 | 23.2 | 45.8 | 70.7 | 89.5 | 13.8 | 39.3 | 59.8 | 70.4 | 85.7 |
| Birth Order Number | |  |  |  |  |  |  |  |  |  |  |
|  | First | 47.8 | 60.9 | 66.5 | 67.7 | 74.7 | 59.0 | 67.7 | 73.3 | 74.8 | 79.7 |
|  | Second | 36.1 | 26.4 | 24.9 | 25.2 | 22.7 | 31.2 | 26.4 | 23.2 | 21.3 | 19.2 |
|  | Third | 16.2 | 12.8 | 8.6 | 7.1 | 2.6 | 9.8 | 5.9 | 3.5 | 3.9 | 1.1 |
| Pregnancy Wanted | |  |  |  |  |  |  |  |  |  |  |
|  | Then | 64.5 | 68.7 | 72.3 | 71.8 | 76.6 | 76.0 | 75.9 | 80.8 | 78.5 | 83.5 |
|  | Later | 15.1 | 15.5 | 15.9 | 17.2 | 15.0 | 12.8 | 14.3 | 13.1 | 13.2 | 11.7 |
|  | No more | 20.4 | 15.8 | 11.8 | 10.9 | 8.4 | 11.3 | 9.8 | 6.1 | 8.4 | 4.8 |
| Currently Working | |  |  |  |  |  |  |  |  |  |  |
|  | No | 90.4 | 91.3 | 90.7 | 88.3 | 89.8 | 47.0 | 55.6 | 63.9 | 68.5 | 79.3 |
|  | Yes | 9.6 | 8.7 | 9.3 | 11.7 | 10.2 | 53.0 | 44.4 | 36.1 | 31.5 | 20.7 |
|  | National | 20.7 | 19.1 | 19.3 | 20.1 | 20.8 | 21.6 | 20.2 | 18.0 | 19.8 | 20.4 |
