# Supplementary material for: Socioeconomic inequality and urban-rural disparity of antenatal care visits in Bangladesh: A trend and decomposition analysis
Source: PLoS One. 2024 Mar 25;19(3):e0301106. doi: 10.1371/journal.pone.0301106 (PMC10962795; doi:10.1371/journal.pone.0301106)
Supplement: S2 Table — (DOCX) [file pone.0301106.s004.docx]

S2 Table. Logistic regression results for the factors associated with 4^+^antenatal care visits and 1^+^antenatal care visits.

|  | | 4^+^ Antenatal Care Visits ($\geq4=1, <4=0$) | | | | 1^+^ Antenatal Care Visits (Yes/No) | | | |
| --- | --- | --- | --- | --- | --- | --- | --- | --- | --- |
|  | | 2011 | | 2017 | | 2011 | | 2017 | |
|  | | AOR  (95% CI) | P  -value | AOR  (95% CI) | P  -value | AOR  (95% CI) | P  -value | AOR  (95% CI) | P  -value |
| Division (Ref: Barisal) | |  |  |  |  |  |  |  |  |
|  | Chittagong | 0.51 [0.35,0.73] | 0.00 | 0.76 [0.55,1.07] | 0.11 | 0.66 [0.48,0.91] | 0.01 | 1.29 [0.82,2.07] | 0.26 |
|  | Dhaka | 0.62 [0.44,0.87] | 0.01 | 1.02 [0.73,1.42] | 0.93 | 0.77 [0.56,1.04] | 0.09 | 1.23 [0.74,2.07] | 0.41 |
|  | Khulna | 0.76[0.54,1.07] | 0.11 | 1.61 [1.11,2.34] | 0.01 | 1.00 [0.73,1.38] | 0.99 | 2.7 [1.28,5.72] | 0.01 |
|  | Mymensingh |  |  | 1.39 [0.95,2.04] | 0.09 |  |  | 1.56[0.87,2.77] | 0.13 |
|  | Rajshahi | 0.72 [0.50,1.02] | 0.06 | 1.18 [0.83,1.69] | 0.36 | 1.27 [0.89, 1.82] | 0.18 | 2.19 [1.20,4.01] | 0.01 |
|  | Rangpur | 1.89 [1.29,2.78] | 0.00 | 2.64 [1.79,3.88] | 0.00 | 1.89 [1.33,2.71] | 0.00 | 3.53 [1.66,7.52] | 0.00 |
|  | Sylhet | 0.53[0.35,0.79] | 0.00 | 0.98 [0.66,1.44] | 0.89 | 0.56 [0.40,0.77] | 0.00 | 1.19 [0.73,1.94] | 0.48 |
| Place of Residence (Ref: Urban) | |  |  |  |  |  |  |  |  |
|  | Rural | 0.53 [0.43,0.65] | 0.00 | 0.74 [0.60,0.91] | 0.00 | Insignificant | | | |
| Women’s Education (Ref: No) | |  |  |  |  |  |  |  |  |
|  | Primary | 1.49 [1.12,1.99] | 0.01 | 1.82 [1.31,2.55] | 0.00 | 1.54 [1.28,1.87] | 0.00 | 1.93 [1.39,2.69] | 0.00 |
|  | Secondary | 2.07 [1.57,2.72] | 0.00 | 2.35 [1.69,3.25] | 0.00 | 2.33 [1.87,2.92] | 0.00 | 2.50 [1.68,3.73] | 0.00 |
|  | Higher | 3.85 [2.64,5.64] | 0.00 | 2.68 [1.83,3.93] | 0.00 | 7.15 [4.02,12.74] | 0.00 | 4.77 [1.76,12.8] | 0.00 |
| Last Birth C-Section (Ref: No) | |  |  |  |  |  |  |  |  |
|  | Yes | 2.10 [1.73,2.55] | 0.00 | 2.03 [1.74,2.37] | 0.00 | 2.68 [2.06,3.49] | 0.00 | 4.00 [2.15,7.43] | 0.00 |
| Partner’s Education (Ref: No) | |  |  |  |  |  |  |  |  |
|  | Primary | 1.09 [0.87,1.38] | 0.42 | 1.03 [0.81,1.31] | 0.81 | 1.11 [0.93,1.31] | 0.24 | 1.00 [0.72,1.39] | 0.99 |
|  | Secondary | 1.36 [1.08,1.72] | 0.01 | 1.31 [1.02.1.69] | 0.03 | 1.31 [1.07,1.60] | 0.01 | 1.58 [1.04,2.39] | 0.03 |
|  | Higher | 1.78 [1.33,2.39] | 0.00 | 1.99 [1.44,2.75] | 0.00 | 1.63 [1.19,2.25] | 0.00 | 2.34 [0.98,5.59] | 0.05 |
| Wealth Status (Ref: Poorest) | |  |  |  |  |  |  |  |  |
|  | Poorer | 1.09 [0.81,1.46] | 0.57 | 1.04 [0.84,1.29] | 0.72 | 0.90 [0.75,1.09] | 0.28 | 1.30 [0.93,1.82] | 0.12 |
|  | Middle | 1.36 [1.03,1.79] | 0.03 | 1.29 [1.02,1.62] | 0.03 | 1.26 [1.04,1.52] | 0.02 | 1.63 [1.08,2.45] | 0.02 |
|  | Richer | 1.69 [1.27,2.26] | 0.00 | 1.44 [1.12,1.86] | 0.01 | 1.93 [1.53,2.43] | 0.00 | 2.29 [1.45,3.62] | 0.00 |
|  | Richest | 2.84 [2.04,3.97] | 0.00 | 2.36 [1.72,3.24] | 0.00 | 4.29 [3.09,5.94] | 0.00 | 5.09 [2.29,11.28] | 0.00 |
| Watching TV (Not at all) | |  |  |  |  |  |  |  |  |
|  | Less than once a week | 1.06 [0.84,1.32] | 0.63 | 1.34 [1.03,1.73] | 0.02 | 1.12 [0.92,1.36] | 0.26 | 1.28 [0.83,1.96] | 0.26 |
|  | At least once a week | 1.38 [1.15,1.66] | 0.00 | 1.42 [1.19,1.69] | 0.00 | 1.37 [1.15,1.62] | 0.00 | 1.50 [1.08,2.09] | 0.02 |
| Birth Order Number (Ref: First) | |  |  |  |  |  |  |  |  |
|  | Second | 0.81 [0.68,0.96] | 0.02 | 1.02 [0.86,1.22] | 0.81 | 0.79 [0.68,0.93] | 0.00 | 0.71 [0.54,0.94] | 0.02 |
|  | Third | 0.44 [0.29,0.66] | 0.00 | 0.64 [0.41,0.99] | 0.04 | 0.65 [0.53,0.79] | 0.00 | 0.55 [0.37,0.81] | 0.00 |
| Pregnancy Wanted (Ref: Then) | |  |  |  |  |  |  |  |  |
|  | Later | 1.01 [0.85,1.19] | 0.90 | 0.75 [0.62,0.92] | 0.01 | Insignificant | | | |
|  | No more | 0.92 [0.69,1.20] | 0.52 | 0.67 [0.51,0.87] | 0.00 |  |  |  |  |
| Currently Working (Ref: No) | |  |  |  |  | Insignificant | | | |
|  | Yes | 0.89 [0.69,1.32] | 0.32 | 1.22 [1.03,1.44] | 0.02 |  |  |  |  |
