# Supplementary material for: Socioeconomic inequality and urban-rural disparity of antenatal care visits in Bangladesh: A trend and decomposition analysis
Source: PLoS One. 2024 Mar 25;19(3):e0301106. doi: 10.1371/journal.pone.0301106 (PMC10962795; doi:10.1371/journal.pone.0301106)
Supplement: S3 Table — (DOCX) [file pone.0301106.s005.docx]

S3 Table. Decomposition of Erreygers concentration index for 1^+^antenatal care visits (Yes/No), 2011-2017.

|  | | Marginal Effects  & p-value | | | | Marginal Effects  *Mean | | ECI | | Contribution to CI | | Contribution to CI (%) | | |
| --- | --- | --- | --- | --- | --- | --- | --- | --- | --- | --- | --- | --- | --- | --- |
|  | | 2011 | | 2017 | | 2011 | 2017 | 2011 | 2017 | 2011 | 2017 | 2011 | 2017 | Change |
| Division (Ref: Barisal) | |  |  |  |  |  |  |  |  |  |  |  |  |  |
|  | Chittagong | -0.075 | 0.01 | 0.021 | 0.26 | -0.016 | 0.004 | 0.060 | 0.071 | -0.004 | 0.001 | -1.04 | 1.00 | 2.03 |
|  | Dhaka | -0.049 | 0.09 | 0.017 | 0.42 | -0.015 | 0.004 | 0.105 | 0.264 | -0.006 | 0.005 | -1.71 | 3.73 | 5.44 |
|  | Khulna | 0.000 | 0.99 | 0.063 | 0.01 | 0.000 | 0.006 | 0.031 | 0.006 | 0.000 | 0.000 | 0.00 | 0.11 | 0.11 |
|  | Mymensingh |  |  | 0.034 | 0.13 |  | 0.003 |  | -0.075 |  | -0.001 |  | -0.67 | -0.67 |
|  | Rajshahi | 0.042 | 0.18 | 0.054 | 0.01 | 0.006 | 0.006 | -0.052 | -0.036 | -0.001 | -0.001 | -0.32 | -0.71 | -0.39 |
|  | Rangpur | 0.106 | 0.00 | 0.074 | 0.00 | 0.012 | 0.008 | -0.099 | -0.136 | -0.005 | -0.004 | -1.22 | -3.36 | -2.15 |
|  | Sylhet | -0.109 | 0.00 | 0.014 | 0.48 | -0.007 | 0.001 | -0.008 | -0.036 | 0.000 | 0.000 | 0.07 | -0.12 | -0.19 |
| Women’s Education (Ref: No) | |  |  |  |  |  |  |  |  |  |  |  |  |  |
|  | Primary | 0.089 | 0.00 | 0.057 | 0.00 | 0.027 | 0.016 | -0.213 | -0.282 | -0.023 | -0.018 | -6.05 | -13.99 | -7.94 |
|  | Secondary | 0.167 | 0.00 | 0.073 | 0.00 | 0.072 | 0.036 | 0.330 | 0.078 | 0.095 | 0.011 | 25.34 | 8.91 | -16.43 |
|  | Higher | 0.329 | 0.00 | 0.103 | 0.01 | 0.025 | 0.018 | 0.181 | 0.286 | 0.018 | 0.020 | 4.81 | 15.95 | 11.15 |
| Last Birth C-Section (Ref: No) | |  |  |  |  |  |  |  |  |  |  |  |  |  |
|  | Yes | 0.164 | 0.00 | 0.065 | 0.00 | 0.025 | 0.022 | 0.260 | 0.365 | 0.026 | 0.031 | 6.92 | 24.94 | 18.01 |
| Partner’s Education (Ref: No) | |  |  |  |  |  |  |  |  |  |  |  |  |  |
|  | Primary | 0.019 | 0.24 | 0.000 | 0.99 | 0.005 | 0.000 | -0.116 | -0.275 | -0.003 | 0.000 | -0.68 | 0.00 | 0.67 |
|  | Secondary | 0.049 | 0.01 | 0.029 | 0.03 | 0.015 | 0.010 | 0.259 | 0.164 | 0.015 | 0.006 | 4.02 | 5.04 | 1.02 |
|  | Higher | 0.088 | 0.00 | 0.047 | 0.05 | 0.012 | 0.009 | 0.270 | 0.312 | 0.012 | 0.011 | 3.32 | 8.53 | 5.20 |
| Wealth Status (Ref: Poorest) | |  |  |  |  |  |  |  |  |  |  |  |  |  |
|  | Poorer | -0.021 | 0.28 | 0.021 | 0.12 | -0.004 | 0.004 | -0.290 | -0.313 | 0.005 | -0.005 | 1.32 | -4.19 | -5.51 |
|  | Middle | 0.046 | 0.02 | 0.036 | 0.02 | 0.009 | 0.007 | 0.030 | 0.009 | 0.001 | 0.000 | 0.29 | 0.18 | -0.10 |
|  | Richer | 0.127 | 0.00 | 0.054 | 0.00 | 0.025 | 0.011 | 0.340 | 0.329 | 0.034 | 0.014 | 9.06 | 11.40 | 2.34 |
|  | Richest | 0.249 | 0.00 | 0.082 | 0.00 | 0.046 | 0.016 | 0.604 | 0.630 | 0.112 | 0.040 | 29.78 | 31.90 | 2.12 |
| Watching TV (Ref: Not at all) | |  |  |  |  |  |  |  |  |  |  |  |  |  |
|  | Less than once a week | 0.020 | 0.27 | 0.016 | 0.26 | 0.003 | 0.002 | -0.092 | -0.058 | -0.001 | 0.000 | -0.25 | -0.27 | -0.02 |
|  | At least once a week | 0.057 | 0.00 | 0.026 | 0.02 | 0.027 | 0.014 | 0.645 | 0.565 | 0.070 | 0.032 | 18.73 | 25.30 | 6.57 |
| Birth Order Number (Ref: First) | |  |  |  |  |  |  |  |  |  |  |  |  |  |
|  | Second | -0.042 | 0.01 | -0.022 | 0.02 | -0.012 | -0.005 | -0.100 | -0.097 | 0.005 | 0.002 | 1.23 | 1.66 | 0.44 |
|  | Third | -0.081 | 0.00 | -0.044 | 0.01 | -0.008 | -0.002 | -0.116 | -0.060 | 0.004 | 0.001 | 0.94 | 0.41 | -0.54 |
|  | Explained |  |  |  |  |  |  |  |  | 0.355 | 0.146 | 94.57 | 115.75 | 21.18 |
|  | Unexplained |  |  |  |  |  |  |  |  | 0.021 | -0.020 | 5.43 | -15.75 | -21.18 |
|  | Total |  |  |  |  |  |  |  |  | 0.376 | 0.126 | 100 | 100.00 |  |
